# A Tutorial Introduction to Heterogeneous Treatment Effect Estimation with Meta-learners

**DOI:** 10.1007/s10488-023-01303-9

**Published:** 2023-11-03

**Authors:** Marie Salditt, Theresa Eckes, Steffen Nestler

**Affiliations:** Institut für Psychologie, University of Münster, Fliednerstr. 21, 48149 Münster, Germany

**Keywords:** Treatment effect heterogeneity, Individual treatment effects, Machine learning, Meta-learners, Causal inference, Personalized medicine

## Abstract

Psychotherapy has been proven to be effective on average, though patients respond very differently to treatment. Understanding which characteristics are associated with treatment effect heterogeneity can help to customize therapy to the individual patient. In this tutorial, we describe different *meta-learners*, which are flexible algorithms that can be used to estimate personalized treatment effects. More specifically, meta-learners decompose treatment effect estimation into multiple prediction tasks, each of which can be solved by any machine learning model. We begin by reviewing necessary assumptions for interpreting the estimated treatment effects as causal, and then give an overview over key concepts of machine learning. Throughout the article, we use an illustrative data example to show how the different meta-learners can be implemented in R. We also point out how current popular practices in psychotherapy research fit into the meta-learning framework. Finally, we show how heterogeneous treatment effects can be analyzed, and point out some challenges in the implementation of meta-learners.

**Supplementary Information:**

The online version contains supplementary material available at 10.1007/s10488-023-01303-9.

## Introduction

In the last decades, clinical psychologists conducted many randomized controlled trials and observational studies to test the effectiveness of psychotherapy. In almost all of these studies, the parameter of interest was the average treatment effect, a measure of the overall impact of treatment, and the results showed that psychotherapy is (on average) efficacious for reducing clinical symptoms (see, e.g., Cuijpers et al., 2020, for depression, Baker et al., 2021 for anxiety disorders, or Kline et al., 2018 for posttraumatic stress disorders). However, researchers and practitioners have long realized that psychotherapy can affect different patients in different ways (see e.g., Kaiser et al., 2022), and knowing patient attributes that are related to such treatment effect heterogeneity is essential to the famous question of “what works for whom” Paul (1967).

To answer this question, clinical psychologists need statistical approaches that allow them to detect subgroups of patients which respond differently to the treatment(s) under consideration. A simple statistical approach involves forming subgroups of participants according to their values in a particular attribute (e.g., using participants’ age to categorize them into young, middle-aged, and old persons). Within each subgroup, the *conditional average treatment effect* (CATE, the respective average treatment effect for the young, middle-aged, and old persons) is estimated, and if the resulting CATEs differ across subgroups, the respective attribute is said to modify the treatment effect (e.g., Wendling et al., 2018a). Such an approach is *theory*-driven because the attributes to form subgroups are specified a priori (see e.g., Hu, 2023, for other theory-based approaches to estimate heterogeneous treatment effects).

Theory-based approaches cannot be employed when one does not know the attributes that define relevant subgroups. In this case, researchers could test every possible attribute combination by including, for example, all interaction terms in a classical linear regression model. However, this approach is not feasible when the number of potential attributes is large, because then the resulting statistical models would contain many parameters (potentially larger than the size of the used sample), which can impair parameter estimation. As a remedy, one can employ *data*-driven covariate selection strategies (e.g., Huibers et al., 2015; Wester et al., 2022. Yet, even with these strategies, the underlying assumption of the classical regression model remains a linear functional relationship between the covariates and the outcome - an assumption that might be violated in the population.

To address this, statistical research suggested several other data-driven approaches that use flexible machine learning methods to estimate a treatment effect for each person based on their covariate values. Heuristically, these approaches can be distinguished into two groups: The first group consists of estimators that are based on altering a specific machine learning method in such a way that it estimates the CATE directly. This group includes methods such as the causal tree (Athey & Imbens, 2016), the causal forest (Athey et al., 2019), causal boosting (Powers et al., 2018), and the Bayesian causal forest (Hill, 2011; Hahn et al., 2020). The second group consists of general algorithms that decompose CATE estimation into multiple sub-problems, each of which can be solved by *any* machine learning method (Künzel et al., 2019). These algorithms are called *meta-learners* and include methods such as the T-learner and the X-learner (see Künzel et al., 2019; Wendling et al., 2018a; Bica et al., 2021; Nie & Wager, 2021; Kennedy, 2022). Regardless of which method is used, the results can then be used for further analyses, such as evaluating which covariates are driving the treatment effect heterogeneity, or for predicting individual treatment effects for new patients in order to derive personalized treatment recommendations.

To accommodate the interest of clinical psychologists in heterogeneous treatment effects, this tutorial explains the most common meta-learners and shows how they can be implemented in the statistical software R (R Core Team, 2023). We focus on meta-learners because they are straightforward to implement in standard statistical software and also very flexible by allowing to incorporate standard statistical models (e.g., the generalized linear model) and/or popular machine learning algorithms (e.g., random forests, gradient boosted trees, neural networks) to estimate heterogeneous treatment effects.^1^ Psychotherapy research has increasingly focused on treatment effect heterogeneity and individual treatment recommendations in the past decade (see e.g., Barber & Muenz 1996; Lutz et al., 2006; Wallace et al., 2013; DeRubeis et al., 2014). One popular approach that has been applied in various therapy studies (e.g., Huibers et al., 2015; Deisenhofer et al., 2018; Keefe et al., 2018; Delgadillo & Gonzalez Salas Duhne, 2020 van Bronswijk et al., 2021; Schwartz et al., 2021) is the personalized advantage index introduced by DeRubeis et al. (2014), which is a measure of the predicted advantage of one therapy relative to another. As we show below, this approach fits well into the meta-learning framework.

Specifically, this tutorial is structured as follows: In Section 2, we introduce the potential outcome framework that we use to define average and conditional average treatment effects and the propensity score. To facilitate understanding of the meta-learners, we then review some machine learning basics in Section 3. In Sect. 4, we describe the data example that we use to illustrate the different meta-learners in the following sections. We then describe the different meta-learners and discuss their strengths and weaknesses in Sect. 5. In Sect. 6, we point out some critical issues in implementing meta-learners. In particular, we explain why and how sample splitting is often implemented when using a meta-learner. Furthermore, we illustrate how to analyze the heterogeneity of treatment effects based on the individual treatment effect estimates obtained from a meta-learner. Throughout the article, we show the R code for implementing the different approaches. Also, because the causal inference and the machine learning literature come with their own terminology that some readers might be unfamiliar with, we provide a glossary at the end of this article.

## Potential Outcome Framework and Heterogeneous Treatment Effects

We consider a setting where the treatment variable *A* is binary (e.g., there is a treatment and a control condition) and the outcome variable *Y* is continuous. For instance, *A* could denote whether participants underwent psychotherapy, and *Y* could denote the symptom severity. For a person *i*, the observed value in the treatment variable is $A_i = 0$ when she belongs to the control group and $A_i = 1$ when she is in the experimental group (of course, *A* could also denote which among two alternative treatments was received, e.g. cognitive-behavioral therapy or psychodynamic therapy, as often the case in current psychotherapy research). Furthermore, several covariate values are available for person *i* (e.g., her age and educational status) that we collect in the vector ${\varvec{X}}_i$. Importantly, we assume that the treatment variable does not influence the covariates. Using the potential outcomes framework (POF; see, e.g., Hernan & Robins, 2020; Imbens & Rubin, 2015 for introductions), we assume that each person has two *potential* outcomes: $Y_i(1)$ denotes the outcome of person *i* if exposed to treatment ($A_i = 1$), and $Y_i(0)$ denotes the outcome of person *i* in absence of treatment ($A_i = 0$). In our example, $Y_i(1)$ would be *i*’s symptom score if she had received psychotherapy, and $Y_i(0)$ would be her score had she not received psychotherapy. Then the individual treatment effect (ITE) $\tau _i$ of person *i* is defined as the difference between the two potential outcomes, $\tau _i = Y_i(1) - Y_i(0)$. We further assume that the observed outcome equals the potential outcome under the treatment level actually received:^2^

1 $$\begin{aligned} Y_i = A_i Y_i(1) + (1-A_i) Y_i(0). \end{aligned}$$

Hence, one can observe only *one* potential outcome value, but never both, with the consequence that the ITE $\tau _i$ cannot be calculated (the *fundamental problem of causal inference*, Holland, 1986).

Statisticians therefore focus on estimating the conditional average treatment effect (CATE) and the average treatment effect (ATE). The CATE $\tau ({\varvec{x}})$ is defined as

2 $$\begin{aligned} \begin{aligned} \tau ({\varvec{x}})&= {\mathbb {E}} [ \tau _i \vert {\varvec{X}}_i = {\varvec{x}}] = {\mathbb {E}} [ Y_i(1) - Y_i(0) \vert {\varvec{X}}_i = {\varvec{x}}] \end{aligned} \end{aligned}$$

where ${\mathbb {E}}$ denotes ‘expectation’ (i.e., the population average). To avoid confusion later, note that the term CATE can refer both to the *function* itself and to the *prediction* of this function at ${\varvec{X}}_i = {\varvec{x}}$, that is, the expected treatment effect for persons with covariate values ${\varvec{x}}$. For instance, we could be interested in the expected treatment effect for persons who are 50 years old and have a university degree (i.e., ${\varvec{x}} = (\textrm{age}, \textrm{education}) = (50, \text {'university degree'})$). If, supposedly, there exists only a single person aged 50 with a university degree in the population, then the CATE of this person equals her ITE. In general, the ITE $\tau _i$ equals the CATE $\tau ({\varvec{x}})$ if all covariates that determine the variability of treatment effects in are included in $X_i$. Thus, estimating the CATE is the best shot at estimating the ITE.

The ATE is the expectation of the CATEs across all covariate value combinations,

3 $$\begin{aligned} \tau&= {\mathbb {E}} [ Y_i(1) - Y_i(0)] \nonumber \\ {}&= {\mathbb {E}} \left[ {\mathbb {E}} [ Y_i(1) - Y_i(0) \vert {\varvec{X}}_i ] \right] = {\mathbb {E}} [\tau ({\varvec{X}}_i)]. \end{aligned}$$

Thus, the ATE is an ‘average’, and if all CATEs are the same, it is said to be homogeneous. By contrast, if the treatment effect varies across persons with different values of the observed covariates, there is treatment effect heterogeneity, and the CATE can be used to identify the subgroups that differ in their treatment effect.

The definition of the ATE and CATE are based on the potential outcomes, and above we stated that some of these values cannot be observed. Therefore, to obtain estimates of the CATE (and the ATE), we have to tie them to the observed values (see Equation (1) above). Furthermore, in observational studies (i.e., where exposure to treatment is non-random), additional assumptions are needed to obtain estimates that can be interpreted as causal. Here, we will rely on *conditional independence* and *positivity*.^3^ Conditional independence states that the potential outcomes are independent of the treatment conditional on the observed covariates (i.e., $\lbrace Y_i(0), Y_i(1) \rbrace \perp A_i \ \vert \ {\varvec{X}}_i$). This entails that all confounding variables were observed and are contained in ${\varvec{X}}_i$. Positivity requires that the conditional probability to receive treatment – the *propensity score* $\pi ({\varvec{x}})$ – is bounded away from 0 and 1:

4 $$\begin{aligned} 0< \pi ({\varvec{x}})&= \text {P}[A_{i} = 1 \vert {\varvec{X}}_{i} \nonumber \\ {}&= {\varvec{x}}] < 1 \quad \text{ for } \text{ all } \ {\varvec{x}} \ \text{ in } \text{ the } \text{ support } \text{ of } \ {\varvec{X}}_i. \end{aligned}$$

This means that for any possible covariate combination, both treated and untreated persons exist. Note that in randomized controlled trials, the propensity score is known by study design (e.g., $\pi ({\varvec{X}}_i) = 0.5$ when treatment groups are of equal size), whereas in observational studies it is unknown and needs to be estimated (see below).

Using the definition of the CATE and the conditional independence and positivity assumption (Hernan & Robins, 2020; Imbens & Rubin, 2015), the CATE can be expressed as

5 $$\begin{aligned} \tau ({\varvec{x}})&= {\mathbb {E}} [ Y_i(1) - Y_i(0) \vert {\varvec{X}}_i = {\varvec{x}}] \nonumber \\ {}&= {\mathbb {E}} [ Y_i \vert {\varvec{X}}_i = {\varvec{x}}, A_i = 1 ] - {\mathbb {E}} [ Y_i \vert {\varvec{X}}_i = {\varvec{x}}, A_i = 0] \nonumber \\ {}&= \mu _1({\varvec{x}}) - \mu _0({\varvec{x}}) \end{aligned}$$

We refer to $\mu _1({\varvec{x}})$ and $\mu _0({\varvec{x}})$ as the conditional mean functions. Note that $\mu _1({\varvec{x}})$ and $\mu _0({\varvec{x}})$ are defined in terms of the observed rather than potential outcomes. Thus, one can estimate the CATE from observed data.

## Machine Learning Basics

Equation 5 shows that an estimate of the CATE (and hence also the ITE) can be obtained when one knows the conditional mean functions $\mu _1({\varvec{x}})$ and $\mu _0({\varvec{x}})$. Estimating such functions is a classical prediction task, for which machine learning methods are well suited. Machine learning refers to any statistical model or algorithm that uses the observed outcome and covariate values to build a model that takes the covariate values as input and predicts the outcome given these covariate values.^4^ When dealing with binary or categorical outcomes, such as determining whether a person receives treatment or not, the prediction concerns a class or category membership and is called classification. When the outcome is continuous, such as measuring the symptom score of a person, the prediction is a real value. This type of prediction is known as regression (that is, the term ’regression’ refers to the prediction of a continuous outcome in general, and ordinary least squares linear regression represents just one among various approaches available for generating such predictions).

In either case, a *training set* is used to build an estimator (or model) of $m({\varvec{x}}) = {\mathbb {E}}[Y_i \vert {\varvec{X}}_i = {\varvec{x}}]$ such that the deviations between the observed (true) outcome values *Y* and the model’s predicted values ${\hat{Y}} = {\hat{m}}({\varvec{x}}) = \hat{{\mathbb {E}}}[Y_i \vert {\varvec{X}}_i = {\varvec{x}}]$ are as small as possible. The magnitude of the deviations is quantified with a *loss function*, and the model’s parameters are estimated in such a way that the value of the loss function is minimal for the training data. To illustrate, a well-known ‘machine learning algorithm’ is the linear regression model, whose predicted values are given by:

6 $$\begin{aligned} {\hat{Y}}_i = \hat{{\mathbb {E}}}[Y_i \vert {\varvec{X}}_i ] = \beta _0 + \beta _1 X_{i1} + \ldots + \beta _p X_{ip}. \end{aligned}$$

The regression coefficients, $\beta _0, \beta _1, \ldots , \beta _p$, are obtained such that they minimize the average of the squared error terms (i.e., the mean squared error [MSE])

7 $$\begin{aligned} MSE(\varvec{\beta }) = \frac{1}{n} \sum _{i=1}^{n} (Y_i - {\hat{Y}}_i)^2. \end{aligned}$$

Thus, the MSE serves as a loss function for the linear regression model. In fact, the MSE is the standard loss function for regression tasks.

Having constructed the prediction model (e.g., having fit the regression model to training data), its performance, that is, its prediction error, has to be assessed on an independent *test set*. It is important to use independent samples for training and evaluating the model because it is likely that the model predicts the outcome values for the training data very well, but only poorly for new (test) data. Thus, if one used the training data to evaluate the model’s predictive performance again, the resulting error estimate would likely be overly optimistic. This phenomenon is called *overfitting* and occurs because the model partly captures irrelevant, random deviations in the training data, which has the consequence that the model does not generalize to new data (see McNeish, 2015; Nestler & Humberg 2022).

As stated above, linear regression is a machine learning algorithm. However, for many prediction tasks it is not the best modeling option, because linear regression presumes a linear relationship between the covariates and the outcome. Hence, the linear regression model provides poor predictions if the true relationship is nonlinear. Furthermore, the model yields unstable parameter estimates when the number of covariates is large relative to the sample size (i.e. when the setting is high-dimensional). More “typical” machine learning methods such as lasso regression, gradient boosted trees, and neural networks (see Hastie et al., 2009 for a thorough overview) are more flexible in this regard because they make fewer or no assumptions about the functional form and can also be applied in high-dimensional settings.

### Random Forests and Cross-validation

**Fig. 1:**
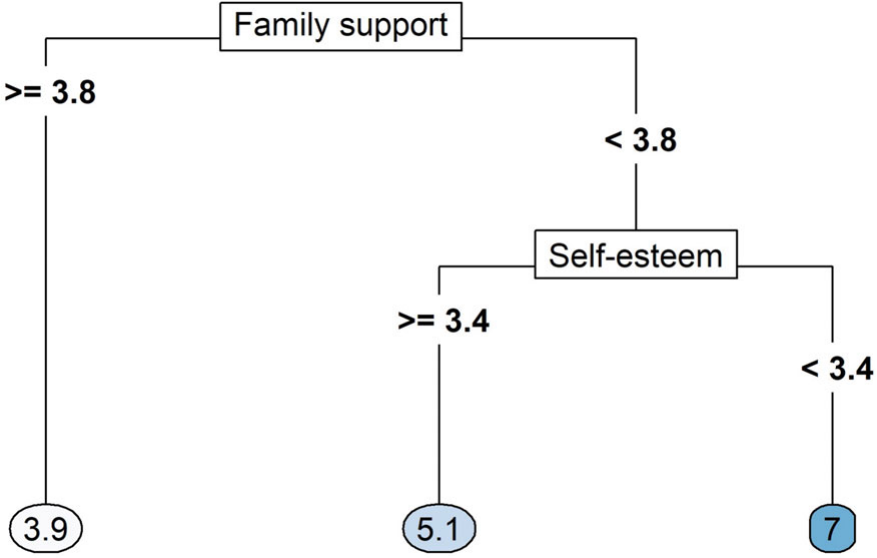
Examplaric regression tree. *Note.* A regression tree to predict a depressive symptom score based on perceived family support and self-esteem (estimated on a 5-point Likert scale). The tree consists of two splits, resulting in three leaves. The lowest symptom score (i.e., 3.9) is predicted for adolescents who rate their family support above 3.8 points. For adolescents who rate their family support lower than 3.8 points, the predicted symptom score further depends on self-esteem. The highest symptom score (i.e., 7) is predicted for adolescents with lower family support and self-esteem. The plot was created with the R package rpart.plot (Milborrow, 2022)

Another machine learning method that performed well in a number of contexts, and that we use throughout the article, is the random forest (Breiman, 2001). A random forest can be used both for classification and regression problems and consists of a collection of decision trees. A single decision tree successively splits the covariate space into disjoint subgroups of persons (the ‘*leaves*’), such that within leaves, the persons are as similar as possible regarding the outcome variable. Then all persons falling into a given leaf obtain the same predicted value. Figure 1 presents an example of a regression tree.

The covariates and covariate values used for splitting are chosen such that the prediction error in the training set is minimized. In a regression tree, for example, the mean of the outcome values in a leaf is used as the predictive value for that leaf, and the splits are found by minimizing the MSE. Note that some variables might not be part of the final tree. Specifically, when a variable is not very predictive of the outcome in the training set, splitting on it will not help decrease the MSE, so the variable will never be chosen for splitting. Thus, unlike a linear regression model, the final decision tree might not contain all covariates. Due to this internal variable selection, a decision tree is better at handling many predictor variables than linear regression.

As stated, the random forest is a collection of trees and computes predictions by averaging the predictions from multiple trees. To obtain good predictions, two tweaks are used when constructing the single trees. First, each tree is fitted on a random subsample of the training set (which is usually obtained via bootstrapping with replacement). Second, at each split only a random subset of the covariates is considered as potential split variables. This has the consequence that a random forest provides more stable predictions than a single tree.

**Fig. 2:**
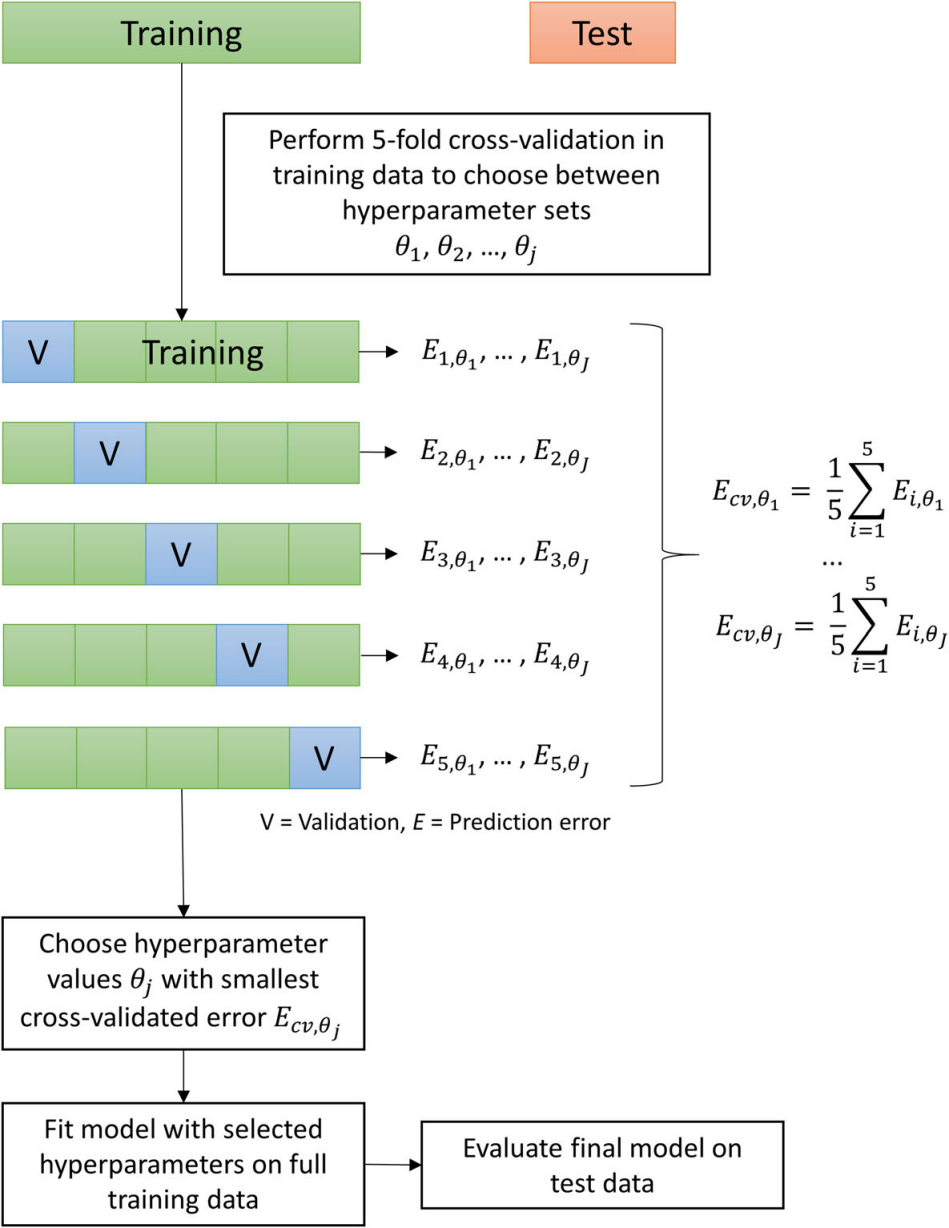
Workflow of tuning and testing machine learning models.

The performance of a random forest depends – amongst other things – on the number of potential covariates considered at each split (henceforth referred to as ‘mtry’), the number of trees in the forest, and the depth of the single trees (the tree depth limits the maximal number of leaves). Such parameters – parameters whose values affect the way the model is built – are called *hyperparameters* in the machine learning literature, and they have to be fixed at specific values before training the model. Unfortunately, researchers do not know a priori which hyperparameter values work best for the problem at hand. Therefore, one tries out several possible hyperparameter values and then selects the ones with the best predictive performance. This process of tuning the hyperparameters is an integral part of building a machine learning model, and the standard approach for doing this is *k*-fold cross-validation (see Figure 2 for an illustration).

Assume that only three mtry values are considered (e.g., 3, 4, and 5) in hyperparameter tuning. To use *k*-fold cross-validation for choosing between these three values, the *training data* is randomly split into *k* equally sized subsets called *folds*. Cross-validation then iterates through these folds: In each of the *k* iterations, one of the *k* subsets is held out as validation data, while the other $k-1$ subsets are used for training the three random forest models, one model for each mtry value. That is, in each iteration, the $k-1$ training subsets are used to fit the models, and the prediction error of each model is calculated on the hold-out dataset. Finally, the prediction errors for each mtry value are separately averaged across the *k* iterations, and the mtry value with the lowest mean prediction error is selected as the final hyperparameter.

Most machine learning software implements hyperparameter tuning via cross-validation, such that the researcher only needs to specify which hyperparameters to tune and how many folds to use.^5^ Furthermore, *k* is typically set to either 5 or 10, because simulation studies found these values to work well (Hastie et al., 2009). In general, however, *k* should be chosen such that each fold is large enough to be representative of the full sample. Finally, after cross-validation, one fixes the hyperparameters to the selected values, refits the model using the whole training dataset, and uses the resulting model to calculate the prediction error on the test set.

**Fig. 3:**
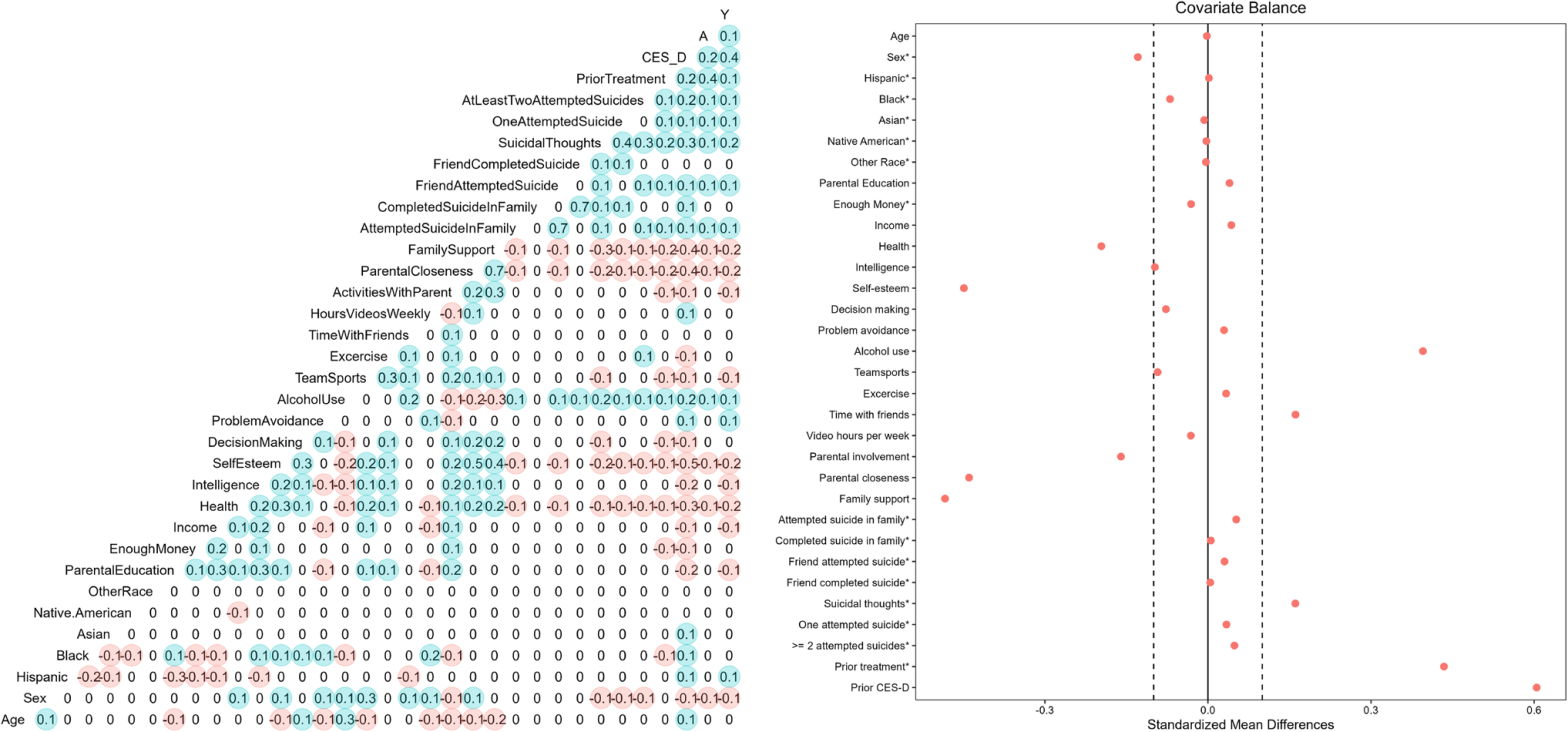
Pairwise correlations and initial covariate imbalance in the illustrative data example. *Note.* Pairwise correlations of all variables in the illustrative data example (left panel) and the covariates’ mean differences between adolescents receiving ($A = 1$) vs. not receiving ($A = 0$) psychological or emotional counseling (right panel). In the case of binary variables (indicated by asterisks), the raw (rather than standardized) mean differences are displayed. The dashed lines indicate the threshold of 0.1 for an acceptable covariate balance. The balance plot was created with the R package cobalt (Greifer, 2022)

## Illustrative Data Example

To illustrate the different meta-learners, we use the public-use datasets of the National Longitudinal Study of Adolescent to Adult Health (Add Health; Harris & Udry, 2022). Add Health is a longitudinal study of a nationally representative sample of 20,000 adolescents aged 12 to 19 during the 1994-95 school year. Since then, the respondents were followed into adulthood with five waves, most recently in 2016-18. We use the Add Health data from wave I (1995), wave II (1996), and wave III (2001-02) to investigate the effect of receiving psychological or emotional counseling on depressive symptoms.

Specifically, we use the answer to the question “In the past year, have you received psychological or emotional counseling?” from the wave II survey as the treatment variable (i.e., $A_i = 1$ if the respondent received counseling, and $A_i = 0$ otherwise). Our outcome of interest, $Y_i$, is the total score on the 9-item-subscale of the CES-Depression scale (CES-D) in the wave III survey. The maximal possible score is 27, ranging from 0 to 25 in our sample ($M = 4.52$, $SD = 4.06$). We control for 25 covariates in total, all of which were assessed before the treatment variable in the wave I survey. Specifically, we include six socio-demographic variables: age, sex (0 = ’female’ and 1 = ’male’), race (Hispanic, White, Black, Native American/Indian, Asian, and other), parental education (0-8, with higher values indicating higher levels of education ), parental income, and whether the respondent’s parents agreed to have enough money to pay their bills (0 = ‘yes’, 1 = ‘no’). Regarding the family setting, we control for parental involvement (measured by the number of shared parent-child activities within the past four weeks, Sieving et al.,^2001^), perceived parental closeness, and perceived family support (1-5, with higher values indicating higher closeness and support, respectively; LeCloux et al., 2016). We further control for several personality and health-related variables as well as for weekly activities, namely self-rated intelligence (6-point Likert scale from 1 = ’moderately below average’ to 6 = ’extremely above average’), health (5-point Likert scale from 1 = ’excellent’ to 5 = ’poor’), self-esteem (0-5, the mean score on 6 items such as “You like yourself just the way you are”), how much the respondent has an analytic approach towards decision making (0-5, the mean score on 5 items such as “When making decisions, you generally use a systematic method for judging and comparing alternatives”), how much the respondent tends to avoid dealing with problems (0-5, the score on the item “You usually go out of your way to avoid having to deal with problems in your life”), alcohol use (1-8, with 1 indicating 2-3 drinks in lifetime and 8 indicating that the respondent drank almost every day in the past 12 months; Sieving et al., 2001), how many times the respondent participated in team sports, did exercise, and spent time with friends during the last week (each measured on a 5-point Likert scale from 0 = ’not at all’ to 5 = ’5 or more times’), and the total hours that the respondent spent with television, videos, or video games. Furthermore, we control for whether the respondent seriously thought about committing suicide (0 = ‘no’, 1 = ‘yes’) and whether a suicide was attempted during the past year (’no attempt’, ’one attempt’, ’two or more attempts’), as well as for a family and a friend suicide composite representing suicide attempts and completion in the past year among family members and friends, respectively (’no attempt’, ’attempted suicide’, ’completed suicide’). Finally, we include prior treatment and prior CES-D score as covariates. We used the R-package caret to impute missing values via bagged trees (Kuhn, 2022). The total sample entailed $n = 3,491$ persons, out of which 353 persons received treatment (i.e., received psychological or emotional counseling). The supplementary material provides a detailed script showing how we formed the sample.

Figure 3 displays the pairwise correlations between the variables (left panel) as well as the mean differences of the covariates between the treatment and control group (right panel). Typically, standardized mean differences below 0.1 are deemed acceptable, whereas covariates with standardized mean differences $\ge 0.1$ are considered to be *imbalanced* (Leite, 2016). As can be seen, several covariates are substantially imbalanced: On average, adolescents who did vs. did not receive counseling had more often already received counseling, had been more depressive and more suicidal in the year before, had consumed more alcohol, had felt less supported by their family and less close to their parents, had spent more time with friends, had rated their self-esteem and health as lower, and were more often female.

We point out that the main purpose of this example is to illustrate the different meta-learners, rather than to draw any substantive conclusions. For example, the validity of the results is limited by the fact that the treatment is not well-defined (i.e., the treatment variable captured whether respondents received *any* psychological or emotional counseling, whose content and quality likely varied to a great extent) and because there might be relevant confounders that we do not control for (e.g., adverse childhood experiences).

## Meta-learners for CATE Estimation

Let us now turn to the estimation of the CATE function $\tau ({\varvec{x}})$ using meta-learners (see Equation 5 again). Meta-learners are algorithms that decompose CATE estimation into multiple prediction problems, each of which can be solved by any machine learning model, and then combine the results of these models to obtain ${\hat{\tau }}({\varvec{x}})$ (Künzel et al., 2019). The machine learning method used to solve a prediction problem is called a *base-learner* and in this tutorial, we always use the random forest as base-learner and we fit the forests with the ranger function in the R package ranger (Wright & Ziegler, 2017).^6^ Most of the prediction problems amount to estimating the conditional means of the outcome and the treatment. The latter are referred to as *nuisance functions*, because they are not of primary interest themselves, but are needed to derive ${\hat{\tau }}({\varvec{x}})$. The meta-learners differ in the number of nuisance functions that need to be estimated. Broadly, the meta-learners can be distinguished into the more simple *conditional mean regression methods* and the more complex *pseudo-outcome methods* (Wendling et al., 2018b; Jacob, 2021; Okasa, 2022). Conditional mean regression methods rely on estimating conditional mean functions of the outcome only. The S-Learner and the T-Learner that we describe below belong to this group of meta-learners. Pseudo-outcome methods require more steps and usually incorporate information from the propensity score in order to increase (statistical) efficiency. Specifically, the pseudo-outcome methods first estimate several nuisance functions (e.g., the conditional means of the outcome and the propensity score) and then combine these estimates into a *pseudo-outcome* ${\hat{\psi }}$. The pseudo-outcome ${\hat{\psi }}$ is an initial approximation of the CATE and to obtain a final estimate of ${\hat{\tau }}({\varvec{x}})$, ${\hat{\psi }}$ is regressed on the covariates ${\varvec{X}}_i$.^7^ This pseudo-outcome regression approach is advantageous compared to just using the pseudo-outcome as the CATE estimate, because firstly, it yields a model that maps the covariates on the estimated treatment effect. Thus, when data for a new person is collected, the pseudo-outcome model can be used to obtain a prediction of this person’s CATE, without having to estimate her values on the nuisance functions. Secondly, it serves to regularize and improve the CATE estimate, since pseudo-outcomes can take rather extreme values (especially when the positivity assumption is nearly violated, that is, some estimated propensity scores are close to 0 or 1). We discuss two pseudo-outcome methods, the X-learner and DR-learner, and also the R-learner, which can be regarded as a special kind of pseudo-outcome method.

### Two-model learner (T-learner) and Single-model learner (S-learner)

Equation (5) shows that a straightforward approach to estimate $\tau ({\varvec{x}})$ is to estimate the conditional mean function in absence of treatment $\mu _0({\varvec{x}}) = {\mathbb {E}} [ Y_i \vert {\varvec{X}}_i = {\varvec{x}}, A_i = 0]$ and the conditional mean function under treatment $\mu _1({\varvec{x}}) = {\mathbb {E}} [ Y_i \vert {\varvec{X}}_i = {\varvec{x}}, A_i = 1]$ by fitting separate prediction models to the data of the control group and the treatment group, respectively. For *every* person, both models are used to generate a predicted value, and the difference between these two values is taken as that person’s CATE estimate. Since this algorithm requires separate estimation of the two conditional mean functions, it is called Two- or T-learner. Note that we could have used different base-learners in the two groups. For instance, we could have fit a linear regression model to the data of the control group and a random forest to the data of the experimental group, respectively.
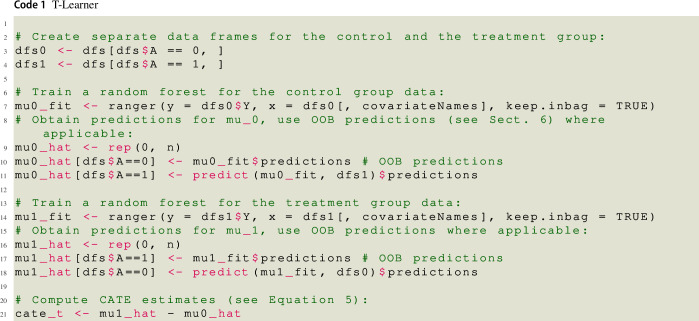


Alternatively, one can use the whole sample to fit a *single* model in which the observed outcome values are modeled as a function of the covariates *and* the treatment indicator variable to obtain ${\hat{\mu }}({\varvec{x}}; a) = \hat{{\mathbb {E}}} [ Y_i \vert {\varvec{X}}_i = {\varvec{x}}, A_i = a]$. The resulting model is then used to generate a prediction for person *i* as if she was in the control group and in the experimental group, respectively. The CATE can then again be estimated by taking the difference between the two predictions. Since a single model is fitted to the data, this algorithm is called Single- or S-learner in the literature. Instead of using a random forest, we could have fit a general linear model to the data, in which the outcome values are regressed on the covariates and the treatment variable indicator. When the S-learner with a general linear model as base-learner is used to obtain an estimate of the ATE, epidemiologists and biostatisticians call this approach the parametric g-formula (Hernan & Robins, 2020). Furthermore, note that the personalized advantage index introduced by DeRubeis et al. (2014) is essentially a CATE estimate obtained by either the S-learner or T-learner.
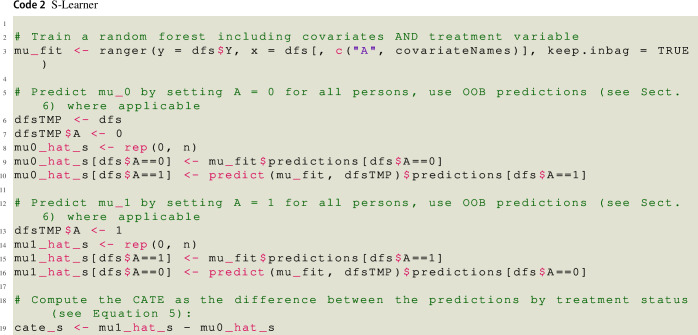


### X-learner

In contrast to the T- and the S-Learner, the X-learner (see Künzel et al., 2019) is a pseudo-outcome method. The first step of the X-learner is identical to the T-learner, that is, one estimates $\mu _1({\varvec{x}})$ and $\mu _0({\varvec{x}})$ separately using the treatment and control group data, respectively. In the second step, the respective missing potential outcome for each person is estimated using ${\hat{\mu }}_1({\varvec{x}})$ and ${\hat{\mu }}_0({\varvec{x}})$, respectively. Then, a difference between the actual observed value and the imputed potential outcome is computed as

8 $$\begin{aligned} {\hat{\psi }}_{X}({\varvec{X}}_i) = {\left\{ \begin{array}{ll} Y_i - {\hat{\mu }}_{0}({\varvec{X}}_i), &{} A_i = 1 \\ {\hat{\mu }}_{1}({\varvec{X}}_i) - Y_i, &{} A_i = 0 \end{array}\right. } \end{aligned}$$

The resulting values are the pseudo-outcomes of the X-learner. They are used to obtain two estimates of the CATE, one for the control group, ${\hat{\tau }}_0({\varvec{x}})$, and one for the treatment group, ${\hat{\tau }}_1({\varvec{x}})$, by separately modeling the pseudo-outcome as a function of the covariates in the control and treatment group, respectively. Finally, the CATE is estimated as a weighted^8^ average of ${\hat{\tau }}_0({\varvec{x}})$ and ${\hat{\tau }}_1({\varvec{x}})$, using the propensity score for weighting:

9 $$\begin{aligned} {\hat{\tau }}({\varvec{x}}) = {\hat{\pi }}({\varvec{x}}){\hat{\tau }}_0({\varvec{x}}) + [1-{\hat{\pi }}({\varvec{x}})]{\hat{\tau }}_1({\varvec{x}}). \end{aligned}$$

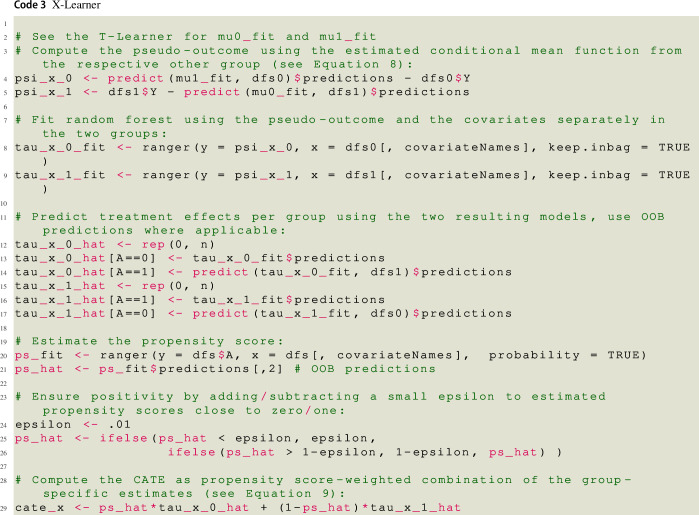


### Doubly-Robust Learner (DR-Learner)

As the X-learner, the DR-learner (see Kennedy, 2022) requires estimating both conditional mean functions separately in the two groups as well as estimating the propensity score. Given these estimates, the pseudo-outcome of the DR-learner is given by

10 $$\begin{aligned} {\hat{\psi }}_{DR}({\varvec{X}}_i)=&{\hat{\mu }}_1({\varvec{X}}_i) - {\hat{\mu }}_0({\varvec{X}}_i) + \frac{A_i \left[ Y_i - {\hat{\mu }}_1({\varvec{X}}_i) \right] }{{\hat{\pi }}({\varvec{X}}_i)} \nonumber \\ {}&-\frac{(1-A_i) \left[ Y_i - {\hat{\mu }}_0({\varvec{X}}_i)\right] }{1-{\hat{\pi }}({\varvec{X}}_i)}. \end{aligned}$$

The pseudo-outcome of the DR-estimator is *doubly-robust* (Robins & Rotnitzky, 1995), that is, it is a consistent estimator of the CATE as long as either the two conditional mean functions or the propensity score model is correctly specified (Lunceford & Davidian, 2004; Knaus, 2022). Thus, ${\hat{\psi }}_{DR}({\varvec{X}}_i)$ should still be a good initial approximation of the CATE even if one fails to find a good approximation of the propensity score, as long as the conditional mean functions are estimated well (and vice verca).

As outlined above, ${\hat{\psi }}_{DR}({\varvec{X}}_i)$ is then regressed on the observed covariates to obtain the DR-learner’s final CATE estimate ${\hat{\tau }}({\varvec{x}})$. A potential drawback of the DR-learner is that extreme, ’unusual’ propensity scores (propensity scores close to zero for treated persons or close to one for untreated persons) can lead to outlying pseudo-outcomes, rendering the DR-estimates unstable (i.e., causing them to be highly variable). The DR-estimator is thus sensitive to near violations of the overlap assumption.
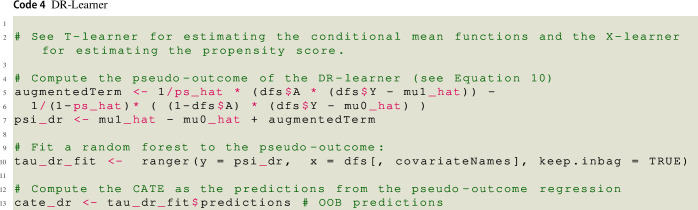


### R-Learner

The final meta-learner that we consider here is the R-learner (see Nie & Wager, 2021). In order to capture treatment effect heterogeneity, the R-learner uses a specific loss function, the so-called R-loss. Minimizing the R-loss is equivalent to fitting a weighted pseudo-outcome regression. Specifically, the R-learner starts with estimating the propensity score and the conditional mean of the outcome given the covariates, $m({\varvec{x}}) = {\mathbb {E}}[Y_i | {\varvec{X}}_i = {\varvec{x}}]$. Then, the CATE is obtained by minimizing

11 $$\begin{aligned} {\hat{L}}_{R}\left[ \tau (\cdot )\right]&= \frac{1}{n} \sum _{i=1}^{n} \lbrace A_i - {\hat{\pi }}({\varvec{X}}_i) \rbrace ^2 \left[ \frac{Y_i - {\hat{m}}({\varvec{X}}_i)}{A_i - {\hat{\pi }}({\varvec{X}}_i)} - \tau ({\varvec{X}}_i) \right] ^2 \end{aligned}$$

12 $$\begin{aligned}&= \frac{1}{n} \sum _{i=1}^{n} \lbrace A_i - {\hat{\pi }}({\varvec{X}}_i) \rbrace ^2 \left[ {\hat{\psi }}_{R}({\varvec{X}}_i) - \tau ({\varvec{X}}_i) \right] ^2 \end{aligned}$$

which is equivalent to regressing the pseudo-outcome ${\hat{\psi }}_{R}({\varvec{X}}_i)$ on the observed covariates, weighted by $\lbrace A_i - {\hat{\pi }}({\varvec{X}}_i) \rbrace ^2$. The pseudo-outcome can be motivated by a semiparametric linear model (Robinson, 1988) that uses the residuals from the regression of $Y_i$ on ${\varvec{X}}_i$ [i.e., $Y_i - m({\varvec{X}}_i)$] and the residuals from the regression of $A_i$ on ${\varvec{X}}_i$ [i.e., $A_i - \pi ({\varvec{X}}_i)$] to control for the potential confounding bias of ${\varvec{X}}_i$. However, similarly to the pseudo-outcome of the DR-learner, it can take extreme values due to the term $A_i - {\hat{\pi }}({\varvec{X}}_i)$ in the denominator (i.e., the pseudo-outcome for treated persons with propensity scores close to one and untreated persons with propensity scores close to zero will be very large in absolute value). The weighting then serves to increase efficiency, as persons with extreme pseudo-outcomes (persons with values $A_i - {\hat{\pi }}({\varvec{X}}_i)$ close to zero) are down-weighted by $\lbrace A_i - {\hat{\pi }}({\varvec{X}}_i) \rbrace ^2$ (Jacob, 2021). In contrast to the other meta-learners described so far, the R-learner can only be used with machine learning methods that allow modification of the loss function by passing the weights $\lbrace A_i - {\hat{\pi }}({\varvec{X}}_i) \rbrace ^2$.^9^ However, this applies to a range of machine learning methods implemented in existing software such as random forest (ranger, Wright & Ziegler, 2017), lasso regression, ridge regression (glmnet, Simon et al., 2011), and gradient boosted trees (xgboost, Chen et al., 2022).
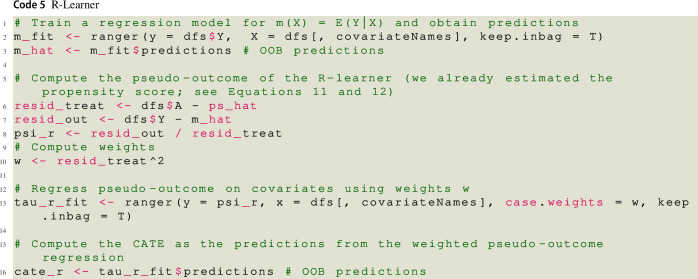


### Comparison of the Different Meta-learners

Having described the most prominent meta-learners, we now compare them with regard to their finite sample properties (see Nie and Wager (2021) and Kennedy (2022) for asymptotic properties of the R-learner and DR-learner, respectively; see Künzel et al. (2019), Curth and van der Schaar (2021), and Okasa (2022) for theoretical and numerical comparisons of the different meta-learners). As to be expected, the relative performance of the different meta-learners (in terms of the MSE) depends on the specific data setting. Also, performance differences are more pronounced the more the group sizes differ and the more confounding is present (i.e., the more the data-generating process deviates from a randomized controlled trial, see e.g., Nie & Wager, 2021; Jacob, 2020), because then it is more important whether and how information from the propensity score is used. Thus, in these cases, pseudo-outcome methods tend to yield better results than the conditional outcome regression models.

The S-learner treats the treatment indicator $A_i$ just as any other covariate when estimating the CATE. Therefore, using the S-Learner in settings where $A_i$ is not very predictive of $Y_i$ can be problematic, because $A_i$ may be omitted as a predictor variable in a fitted machine learning model (e.g., a regression tree might never use $A_i$ for splitting), with the consequence that the CATE cannot be estimated. However, even when the treatment indicator remains in the model, the S-learner may be biased towards zero (see, e.g., Künzel et al., 2019), depending on the amount of regularization of $A_i$ (i.e., the stronger the regularization, the larger the bias).^10^ Nevertheless, in situations where the CATE is simple or indeed zero for many covariate value combinations, the S-learner can work well (Künzel et al., 2019).

In contrast to the S-Learner, the T-learner does not suffer from the regularization problem concerning the treatment variable, because it estimates the conditional mean functions separately in each group. Due to this separate estimation, the T-learner is expected to perform particularly well in situations where the CATE function is more complex than either of the conditional mean functions, as long as both groups are reasonably large. With only few data points available in one of the groups, the T-learner may provide estimates that are unstable and prone to bias, because then it is likely that the estimated conditional mean function overfits the data in the small group such that differences in the two functions are (partly) due to random noise. One can try to avoid this overfitting by using a simple or regularized model, but then the T-learner can suffer from regularization bias. For example, the coefficients of different covariates may be shrinked towards zero in ${\hat{\mu }}_{0}({\varvec{x}})$ and ${\hat{\mu }}_{1}({\varvec{x}})$, such that the T-learner estimates a non-zero CATE even when it is zero everywhere (Nie & Wager, 2021). Thus, in settings with unbalanced treatment group sizes, the T-learner is caught between overfitting and regularization bias, especially when the CATE has a simple form (see Künzel et al. (2019) and Kennedy (2022) for concrete examples in which the T-learner is suboptimal).

The X-learner was developed to overcome the limitations of the S-learner and the T-learner, that is, to work well regardless of whether the CATE has a simple or complex form and despite very different group sizes. This is achieved by using the information of the control group to estimate a conditional treatment effect for the treatment group and vice verca, and then computing the final estimate as (propensity score-) weighted average. The weighting serves to pull the final estimate closer to the estimated effect that relies on the conditional mean function estimated in the larger group (i.e., that is expected to be more accurate).^11^ Similar to the X-learner, the DR-learner and the R-learner estimate the CATE by modelling a pseudo-outcome as a function of the covariates, which can remove some of the bias induced by regularization and overfitting compared to the S-learner and the T-learner (Curth & van der Schaar, 2021). In fact, although the S-learner and T-learner can perform well in particular settings, simulation studies found them to be overall outperformed by the pseudo-outcome methods (Künzel et al., 2019; Kennedy, 2022; Jacob, 2020; Okasa, 2022). Therefore, especially when analysing non-experimental data, psychotherapy researchers should consider to use a pseudo-outcome method rather than the S- or T-learner for CATE estimation.

Comparing the pseudo-outcome methods, it is more difficult to give general considerations apart from the fact that the X-learner is robust towards violation of the positivity assumption due to its different use of the propensity score, whereas the DR-learner, and to a lesser extent also the R-learner, can become unstable in presence of extreme propensity scores (Okasa, 2022).

Okasa (2022) compared the performance of all meta-learners presented in this tutorial in an extensive simulation study, investigating a high-dimensional setting (i.e., 100 covariates, out of which 95 were neither predictive of the outcome nor the treatment variable) with varying complexity of the underlying functions, imbalance of group sizes, and sample size (i.e., $n = 500, 2,000, 8,000$, and 32, 000). Based on the results, he recommends using the X-learner whenever one group makes out 85% or more of the whole sample, irrespective of sample size. In settings where one group makes out 75% of the whole sample, he found the sample size to be decisive: With sample sizes of 500 or 2, 000, the X-learner was still the preferable choice, whereas the DR-learner was favourable with large sample sizes of 8, 000 or greater. When the groups were of equal size, the sample size was less important. Then, the DR-learner and the R-learner were the preferred estimators. However, as a word of caution, these recommendations may change as more simulation studies emerge that examine meta-learners in other settings (e.g., using other data-generating functions).

**Fig. 4:**
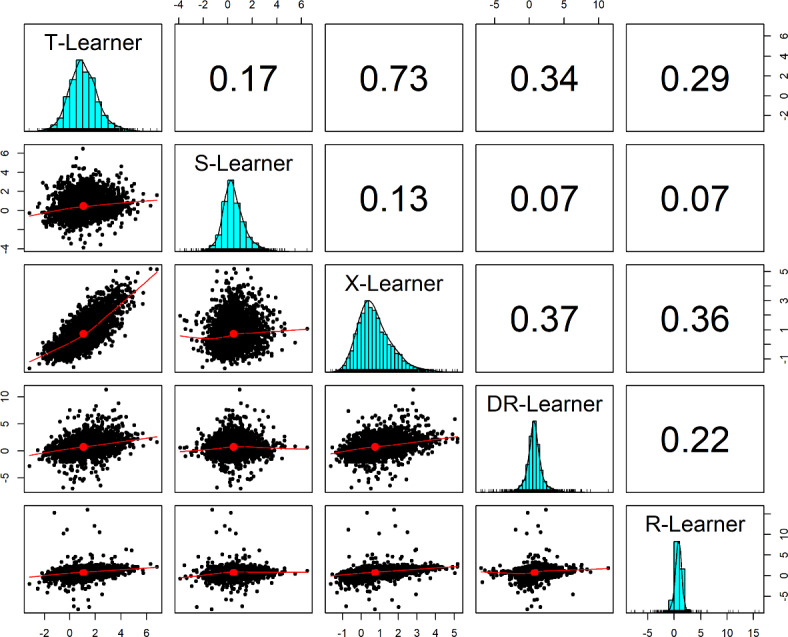
Distribution and pairwise correlations of estimated individual treatment effects for the different meta-learners. *Note.* The plot was created with the R package psych (Revelle, 2022)

Table 1 summarizes the distributions of individual treatment effects as estimated by the five meta-learners in our data example. Histograms and pairwise correlations of the estimated individual treatment effects are displayed in Figure 4.

**Table 1:** Descriptive statistics of the individual treatment effects as estimated by the different meta-learners

	Mean	SD	Min	25%	Median	75%	Max
T-Learner	1.07	1.12	$-$3.19	0.31	0.98	1.75	6.82
S-Learner	0.47	0.93	$-$3.89	$-$0.10	0.36	0.95	6.48
X-Learner	0.74	0.92	$-$1.64	0.10	0.60	1.25	5.19
DR-Learner	0.75	1.25	$-$6.97	0.17	0.69	1.26	11.38
R-Learner	0.75	0.94	$-$8.10	0.38	0.79	1.16	16.04

As can be seen in Table 1, the meta-learners yielded overall similar ATE estimates that range between 0.47 and 1.07 and hence indicate than on average, receiving any kind of psychological or emotional counseling results in a minor increase in depressive symptoms 5 years later (as measured on the 9-item CES-D subscale with a maximum score of 27 points). Further, all meta-learners suggest some treatment effect heterogeneity (the standard deviation ranges from 0.92 for the X-learner to 1.25 for the DR-learner), indicating that the adverse effect of receiving counseling is stronger for some adolescents (with the maximal estimated CATE ranging from 5.19 to 16.04), whereas a small group of adolescents seems to benefit from counseling (i.e., the sign of their estimated treatment effect is negative, with the minimal estimated CATE ranging from $-1.64$ to $-8.10$). Note that although the five meta-learners yield overall similar distributions of estimated CATEs, this does not necessarily imply that the individual estimates are similar as well. Reassuringly, however, the estimated treatment effects are positively correlated across all meta-learners, with the highest correlation between the X-learner and the T-learner (0.73). The S-learner resulted in somewhat different predictions than the other meta-learners, with correlations ranging between 0.07 and 0.17.

Notably, the R-learner (and to a lesser extent also the DR-learner) predict some treatment effects as unreasonably large. This is likely due to the fact that in our data example, only 353 persons underwent counseling, whereas 3, 491 did not. That is, the group sizes were highly unbalanced and the estimated propensity scores were overall very small. In fact, some propensity scores were estimated as 0 and we set values below 0.01 to 0.01 in order to enforce the overlap assumption.^12^ As argued above, this is a setting the X-learner was specifically designed for. Therefore, we focus on the X-learner in the next section,^13^ where we examine how to perform inference on heterogeneous treatment effects (such as testing whether there is evidence for significant treatment effect heterogeneity). Before doing so, however, we point out some key issues with meta-learners.

## Further Issues and Analysis of Treatment Effect Heterogeneity

In the final section of this tutorial, we discuss some further issues to consider when estimating the CATE. We focus on the choice of the base-learners, reducing overfitting via sample splitting and cross-fitting, and the statistical analysis of the CATE estimates.

### Choice of Base-Learners and Model Stacking

The performance of each meta-learner depends upon how well the nuisance functions are estimated (e.g., the conditional mean functions), which in turn hinges upon the choice of the base-learners. For example, Knaus et al. (2021) found the performance of the DR-learner to deteriorate in some settings when using lasso regression as base-learner, whereas it performed relatively well across all settings when the nuisance functions were estimated with a random forest. In practice, one should try to choose a base-learner that is well-suited for the prediction task at hand and to optimize its performance via hyperparameter tuning. We always chose the random forest in the example as base-learner, because it can approximate both simple and complex functions, is comparatively easy to tune, and because previous simulation studies on meta-learners found it to be a good choice (Knaus et al., 2021; Okasa, 2022). Another advantage is that it allows to calculate out-of-bag predictions; a point that we return to in the next subsection.

Nevertheless, it is impossible to know which machine learning method would be the best to use for a given prediction problem, which explains the increased use of the ’Super Learner’ in machine learning applications. The Super Learner is a *model stacking* method. The basic idea of model stacking is to not just use one machine learning method for prediction, but rather to fit several machine learning models to the data (e.g., the generalized linear model, gradient boosted trees, and random forest), and then to combine the predictions of these models. There are different possibilities for combining the predictions, and the Super Learner uses a weighted average, whereby the optimal weights are obtained via cross-validation. It can be shown that (asymptotically) the Super Learner works as well as the best machine learning method included in it (Van der Laan et al., 2007). We refer the reader to Naimi and Balzer (2018) for a more detailed introduction to the Super Learner and for an explanation of how it can be implemented in R.

So far, psychotherapy researchers predominantly used the generalized linear model as base-learner (but see Delgadillo & Gonzalez Salas Duhne, 2020), often selecting covariates beforehand either via covariate selection strategies or via machine learning methods such as the random forest (e.g., Huibers et al., 2015; Webb et al., 2019; Schwartz et al., 2021; van Bronswijk et al., 2021; Senger et al. 2022). The main advantages of using the generalized linear model is that it facilitates interpretation and inference of the CATE: it is straight-forward to assess which covariates are driving the predictions through evaluating significance tests and comparing the (standardized) regression coefficients. With more flexible base-learners, it becomes more difficult to interpret and perform inference on treatment effect heterogeneity, and we will describe approaches for doing so in the next section.

### Sample Splitting and Cross-Fitting

Another important aspect to consider when using meta-learners for estimating the CATE and when subsequently analysing treatment effect heterogeneity is overfitting, which can happen at two points. First, when using pseudo-outcome methods such as the X-, DR-, and R-learner, one estimates some nuisance functions and then uses the (predictions of these) nuisance functions to estimate the CATE in a (weighted) pseudo-outcome regression. However, using the same data to estimate the nuisance functions and the treatment effect function makes the occurrence of overfitting more likely, which in turn can bias the CATE estimator (see e.g. Kennedy, 2022; Chetverikov et al., 2018a). Note that this type of overfitting does not concern the S-and the T-learner, since they only require estimation of the conditional mean function(s) to *compute* the CATE without any further estimation step. The second point concerns the heterogeneity analysis of the estimated treatment effects – which we discuss in the next subsection—and is thus relevant for all meta-learners: Using the same sample for fitting the CATE function and for further analysing the estimated treatment effects can impair the validity of the results. Ideally, one would have access to an independent test set and use a meta-learner’s estimated CATE function, ${\hat{\tau }}({\varvec{x}})$, to obtain the treatment effects for the persons in this test set. Then these estimates would be used to make inferences regarding the treatment effect heterogeneity. In the following, we focus on the first point and describe how different sample splitting approaches can be used to prevent overfitting bias for this case. When turning to the heterogeneity analysis afterwards, we come back to these approaches and discuss how they can be applied in a scenario in which there is no independent test set.

Some machine learning methods have a built-in approach to reduce overfitting as such. A random forest, for instance, is a collection of trees and each tree in the forest is fitted on a bootstrap sample of the training data. As bootstrap samples are random subsamples of the actual sample, not all persons are used when estimating a specific tree in the forest (because some persons are *out-of-bag* (OOB), that is, not part of that tree’s bootstrap sample). This in turn allows to calculate the OOB prediction for a person *i*: The predicted value of *i* is calculated only from the trees that were fitted on bootstrap samples which do *not* contain *i*. Thus, the OOB predictions are, in a certain sense, independent from model fitting, which is why we used OOB predictions throughout the implementations of the meta-learners. However, one might prefer other base-learners, such as gradient boosted trees or a model stacking method like the Super Learner, which do not entail such a built-in approach. A generic approach to prevent overfitting bias is sample splitting (e.g., Chernozhukov et al., 2018a). In the simplest case, the whole sample is randomly split into two sub-samples (or folds), $S_1$ and $S_2$. The first fold $S_1$ is used to train the nuisance functions, whose predictions for the second, independent fold $S_2$ are used to generate the pseudo-outcome. Then the pseudo-outcome is regressed on the covariates in $S_2$, yielding an estimated CATE function.

**Fig. 5:**
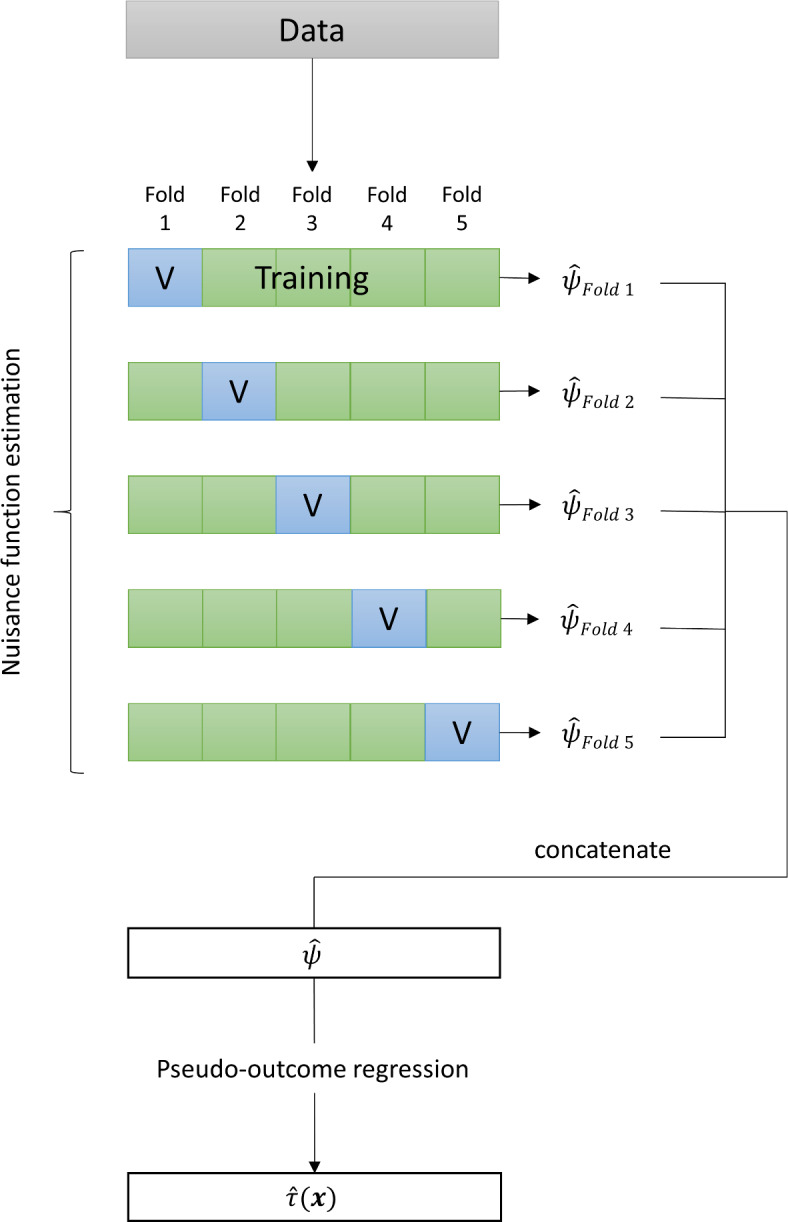
5-fold cross-fitting procedure

A problem of sample splitting is that using only a sub-sample for CATE estimation can result in loss of efficiency and (ironically) underfitting. As a remedy, one typically employs *cross-fitting* (e.g., e.g. Chernozhukov et al., 2018a) to ensure that all the data is used for estimating the CATE function: As before, the nuisance functions are trained in fold $S_1$ and then used to generate the pseudo-outcome for fold $S_2$. Then, the roles of the folds are *reversed* such that the nuisance functions are trained in fold $S_2$ and the results are used to generate the pseudo-outcome for fold $S_1$. As a result, one obtains an ’out-of-fold’ (or cross-fitted) pseudo-outcome for each person *i*, which was calculated based on nuisance functions that did not use person *i* for training. In the final step, this cross-fitted pseudo-outcome is regressed on the covariates in the full sample to obtain ${\hat{\tau }}({\varvec{x}})$. The 2-fold cross-fitting that we just explained can be extended to *k*-fold cross-fitting. In Figure 5 we show a graphical illustration of 5-fold cross-fitting. Using more folds further reduces the risk of underfitting, but at the same time weakens the protection against overfitting. Note that sample splitting (and cross-fitting) should not be mixed-up with cross-validation: While sample splitting is used to separate the estimation of nuisance parameters from estimating the parameter of interest (i.e., the CATE), cross-validation is (mainly) used for hyperparameter tuning of the machine learning method. Thus, in case of the simple sample splitting scheme just explained, cross-validation is done *within* $S_1$ to obtain optimal nuisance functions that are then used in $S_2$ to calculate the pseudo-outcome regression.

Furthermore, we point out that there is another definition of cross-fitting^14^ and that further variants of sample splitting and cross-fitting have been suggested in the literature (see Chernozhukov et al., 2018a and Newey & Robins, 2018, and also Jacob, 2020, and Jacob, 2021, for a discussion of all kinds of splitting approaches). Whichever variant is used, all serve the same goal, that is, to ensure that the nuisance functions used to construct a person’s pseudo-outcome were estimated without using data from that person. However, so far it is unclear which splitting procedure, if at all, is to be preferred in which data setting. Jacob (2020) and Okasa (2022) performed simulation studies to compare the R-learner, DR-learner, and X-learner under different sample splitting schemes, implemented both with and without cross-fitting. Overall, their results indicate that the X-learner usually performs best when using the full sample at all steps (i.e., not splitting the sample at all) and is quite robust under different implementations. In case of the DR-learner and R-learner, it seems to be more relevant whether (and if so, which) sample splitting procedure is used. Furthermore, the results are also dependent on the base-learners that are used. In sum, at present there seems to be no uniformly superior version and we encourage the reader to watch out for forthcoming simulation studies results for further guidance.

### Inference on Heterogeneous Treatment Effects

In the last section, we showed histograms of the CATE estimates for each meta-learner and reported descriptive statistics for the obtained CATE estimates (i.e., the mean, the standard deviation, and the quantiles). Here, we discuss some more recent statistical approaches for making *inference* on features of interest of the CATE (see Chernozhukov et al., 2018b).^15^ Specifically, we describe an overall test for the presence of heterogeneity, how hypotheses regarding subgroup-specific CATEs can be tested (e.g., testing the null hypothesis that the ATE among the 20% most affected persons is zero), and how one can investigate which covariates are associated with treatment effect heterogeneity. In the following, we first focus on the description of these tests (assuming the availability of an independent test set) and present the results for the illustrative data example. At the end of this subsection, we discuss how sample splitting and cross-fitting can be applied to ensure the validity of these tests when there is no independent test set – as was the case in our example – and describe the specific procedure that we implemented here.

**Is there evidence for significant treatment effect heterogeneity?** Chernozhukov et al. (2018b) suggested an overall test for treatment effect heterogeneity and for the quality of a CATE estimator. They focused on randomized controlled trials, but their test can be adjusted for observational data (see Athey et al., 2020; Tibshirani et al., 2023). The (adjusted) test consists of fitting the following regression model:

13 $$\begin{aligned} Y_i - {\hat{m}}({\varvec{X}}_i)=&\ {} \beta _1 \left[ A_i - {\hat{\pi }}({\varvec{X}}_i) \right] \nonumber \\{}&{} + \beta _2 \left\{ \left[ {\hat{\tau }}({\varvec{X}}_i) - {\hat{\tau }} \right] \left[ A_i - {\hat{\pi }}({\varvec{X}}_i) \right] \right\} + \epsilon , \end{aligned}$$

where ${\hat{m}}({\varvec{X}}_i)$ is the mean function estimate of *i*, ${\hat{\pi }}({\varvec{X}}_i)$ is the propensity score estimate, and ${\hat{\tau }} = \frac{1}{n} \sum _{i=1}^{n} {\hat{\tau }}({\varvec{X}}_i)$ is the ATE estimated from the meta-learner’s CATE estimates (i.e., the mean of these estimates, see Table 1). The coefficient $\beta _2$ measures how much the CATE estimates covary with the true CATE. If the meta-learner adequately captures the true heterogeneity, then $\beta _2 = 1$ (Chernozhukov et al., 2018b). Therefore, when $\beta _2$ is significantly greater than zero, this indicates that there is significant treatment effect heterogeneity and that it was captured by the meta-learner at least to some extent. The results for the illustrative data example (using the X-learner) are shown in Table 2 (see the supplementary material for the corresponding R code).

**Table 2:** Results of global test for treatment effect heterogeneity

$\beta _1$	$\beta _2$
0.661	0.943
(0.110, 1.208)	(0.293, 1.590)
[.019]	[.004]

*Note.* Medians over 50 splits. Median confidence intervals ($\alpha = .05$) in parenthesis. P-values for the hypothesis that the parameter is equal to zero against the two-sided alternative in brackets.

The coefficient $\beta _1$ in model (13) equals the ATE (if the true functions $m(\varvec{X}_i), \pi (\varvec{X}_i)$ were used instead of estimates). Thus, in line with the results of the meta-learners, the significant estimate of 0.66 indicates that on average, receiving counseling leads to a slight but significant increase in depressive symptoms. The ATE estimate of the X-learner (0.74, see Table 1) was in a similar range, but indicates that the average prediction of the X-learner is not entirely correct. Furthermore, since $\beta _2$ is significant and estimated close to 1, we can reject the null hypothesis of no treatment effect heterogeneity and infer that the X-learner did a good job at capturing the treatment effect heterogeneity.

**What are the treatment effects across subgroups?** Having seen that there is significant treatment effect heterogeneity, it is interesting to investigate how the treatment effects vary across persons. To this end, we can sort the persons by their estimated CATE, and then split them into subgroups based on quantiles. Here, we split the sample into five subgroups, $G_1, \ldots , G_5$, but note that the number of subgroups is somewhat arbitrary. Thereafter, we fit the following regression model:

14 $$\begin{aligned} Y_i - {\hat{m}}({\varvec{X}}_i) = \left[ A_i - {\hat{\pi }}({\varvec{X}}_i) \right] \sum _{k=1}^{5} \gamma _k D_{k, i} + \epsilon , \end{aligned}$$

where $D_{k, i}$ is a dummy variable for the *k*th subgroup, that is, $D_{k, i}$ is one when the predicted CATE of person *i* is in group $G_k$, and zero otherwise. The parameters of interest in this model are the coefficients $\gamma _k$, which equal the CATE in subgroup *k* (again, if the true functions $m(\varvec{X}_i), \pi (\varvec{X}_i)$ were used): $\gamma _k = {\mathbb {E}}[\tau ({\varvec{X}}_i) \vert G_k]$. These subgroup CATEs are called sorted group average treatment effects (GATES) (see Chernozhukov, Demirer, et al., 2018b, and also Jacob, 2019).^16^ In some cases, the resulting GATES may not be monotonic (although one would expect them to be, since the subgroups were defined based on the predicted strength of treatment effect). Therefore, it is recommended to sort the GATES when using them for further testing, such that they are monotonic. This has the effect that the GATES better approximate the ideal GATES (i.e., the GATES that would be obtained, hypothetically, if the subgroups were defined based on the true CATE).

**Fig. 6:**
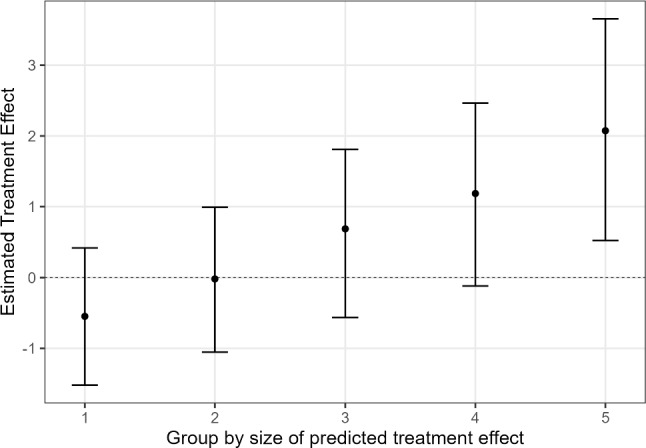
GATES of receiving counseling. *Note.* Median point estimates of treatment effects in subgroups (defined based on the X-learner’s predicted CATE), based on 50 splits. Error bars represent the median 95% confidence intervals

Figure 6 presents the GATES for the illustrative data example. As can be seen, for most of the subgroups, receiving counseling does not have a significant effect on depressive symptoms five years later. However, for the 20% most (adversely) affected adolescents, receiving treatment leads to an average increase of 2 points on the CES-D and this increase is significantly different from zero.

Estimating GATES is just one example of performing a subgroup analysis. In psychotherapy research, it is common to first sort persons based on their estimated CATE into two or three groups: persons for which receiving treatment is indicated (e.g., if a higher outcome indicates more symptoms, persons whose estimated CATE has a negative sign or is lower than some statistical or clinical cut-off), persons for which treatment is not recommended (estimated CATEs with positive sign or higher than the cut-off), and, optionally, persons for which receiving treatment is expected to be neither strongly beneficial nor harmful (estimated CATES around zero). Then, one compares the outcomes between persons who received their model-indicated recommendation (the ’optimal’ group) versus persons who did not (the ’non-optimal’ group) and tests whether the mean outcomes differ significantly (e.g., DeRubeis et al., 2014). If the sample is observational, propensity score methods such as propensity score matching or weighting should be used in order for this comparison to be informative (e.g., Delgadillo & Gonzalez Salas Duhne, 2020). If the outcomes of the optimal group are on average significantly better than the outcomes of the non-optimal group, the estimated CATE function is deemed useful for clinical practice, that is, for informing treatment recommendations for future patients. However, predicting ITEs is a highly challenging task, and as DeRubeis et al. (2014) discuss, the clinical utility of the predictive model should then be tested further in a prospective way.

**Which covariates are associated with the treatment effect heterogeneity?** When the global test and the GATES reveal substantial treatment effect heterogeneity, one seeks to better understand which variables drive the heterogeneity. To this end, one can compare the average (as well as variances, covariances, etc.) of baseline covariates across the subgroups. The comparison of average covariate levels between the most and least affected subgroups is called classification analysis (CLAN; see Chernozhukov, Demirer, et al., 2018b). For the data example, we tested the covariate’s mean differences between the 20% most positively affected and the 20% most negatively affected adolescents with Welch-tests, using the Holm correction to adjust for multiple testing (the R code is provided in the supplementary material). Table 3 presents the results for those covariates which have a non-negligible mean difference between the treatment and untreated adolescents (i.e., the Hedge’s *g* of their absolute mean difference is larger than 0.20). The most pronounced differences at baseline were that the most negatively affected adolescents (the fifth subgroup, whose average effect of counseling is a 2 point increase in depressive symptoms) on average spend less time with friends, drink less alcohol and do less exercise, have a higher tendency to avoid problems, and feel more supported by their family. Note that the differences in baseline covariates between subgroups cannot be interpreted as causal (e.g., we cannot infer that consuming less alcohol will negatively influence the effect of receiving counseling), but might help to shed light on the true factors underlying heterogenous treatment effects.

**Table 3:** Results of classification analysis

	$20\%$ Most Positively Affected	$20\%$ Most Negatively Affected	Difference	
	$M_{G_1}$ (CI)	$M_{G_5}$ (CI)	$M_{G_1} - M_{G_5}$ (CI)	Hedge’s g
Hispanic	.05	.14	**−0.09**	−0.32
	(.04, .07)	(.12, .17)	(−0.12, −0.06)	
Black	.12	.25	**−0.13**	−0.34
	(.10, .14)	(.22, .28)	(−0.17, −0.09)	
Asian	.00	.09	−0.9	−0.44
	(.00, .01)	(.07, .11)	(−0.11, −0.07)	
Health	4.05	3.68	**0.36**	0.41
	(3.99, 4.11)	(3.62, 3.75)	(0.27, 0.45)	
Problem avoidance	2.87	3.40	**−0.53**	−0.52
	(2.80, 2.94)	(3.33, 3.47)	(−0.64, −0.43)	
Alcohol use	2.95	1.99	**0.96**	0.57
	(2.81, 3.08)	(1.89, 2.09)	(0.79, 1.13)	
Teamsports	1.52	1.20	**0.32**	0.29
	(1.44, 1.60)	(1.12, 1.28)	(0.21, 0.43)	
Excercise	1.91	1.37	**0.54**	0.54
	(1.84, 1.98)	(1.30, 1.44)	(0.44, 0.64)	
Time with friends	2.50	1.09	1.41	1.74
	(2.45, 2.55)	(1.03, 1.15)	(1.33, 1.49)	
Video hours per week	19.54	25.47	**−5.94**	.0.27
	(17.96, 21.12)	(23.98, 26.97)	(−8.11, −3.76)	
Parental involvement	5.26	6.62	**−1.36**	−0.41
	(5.02, 5.50)	(6.39, 6.84)	(−1.69, −1.03)	
Parental closeness	4.20	4.45	**−0.24**	−0.39
	(4.16, 4.25)	(4.41, 4.49)	(−0.31, −0.18)	
Family support	3.78	4.14	**−0.35**	−0.50
	(3.73, 3.83)	(4.09, 4.18)	(−0.43, −0.28)	
$\ge 2$ attempted suicides	.05	.01	**.05**	0.28
	(.04, .07)	(.00, .01)	(.03, .06)	
Prior treatment	.17	.08	**0.10**	0.29
	(.14, .20)	(.06, .09)	(0.06, 0.13)	
Prior CES−D	10.39	12.18	**−1.79**	−0.23
	(9.79, 10.99)	(11.67, 12.69)	(−2.58, −1.01)	

*Note.* Medians over 50 splits. $M_{G_1}$ = mean in first subgroup; $M_{G_5}$ = mean in fifth subgroup.

Confidence intervals ($\alpha = .05$) in parenthesis. Significant differences are shown in bold (p-values were adjusted for multiple testing using Holm’s correction).

**Fig. 7:**
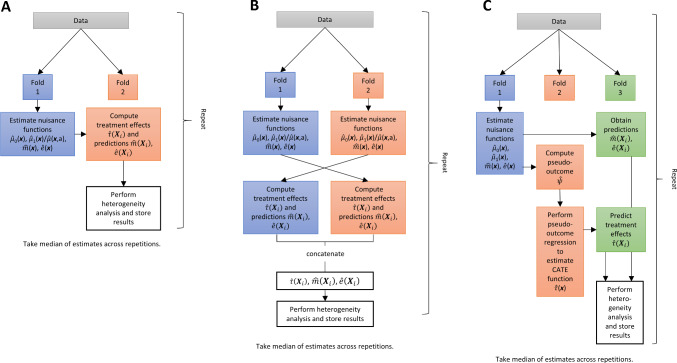
2-fold sample splitting (**A**), 2-fold cross-fitting (**B**), and 3-fold sample splitting (**C**) procedure for separating the estimation of the CATE function from the heterogeneity analysis. *Note.* Procedure C can only be applied to pseudo-outcome methods, where the additional split aims at preventing overfitting due to using the same data for nuisance function estimation and pseudo-outcome regression

**Fig. 8:**
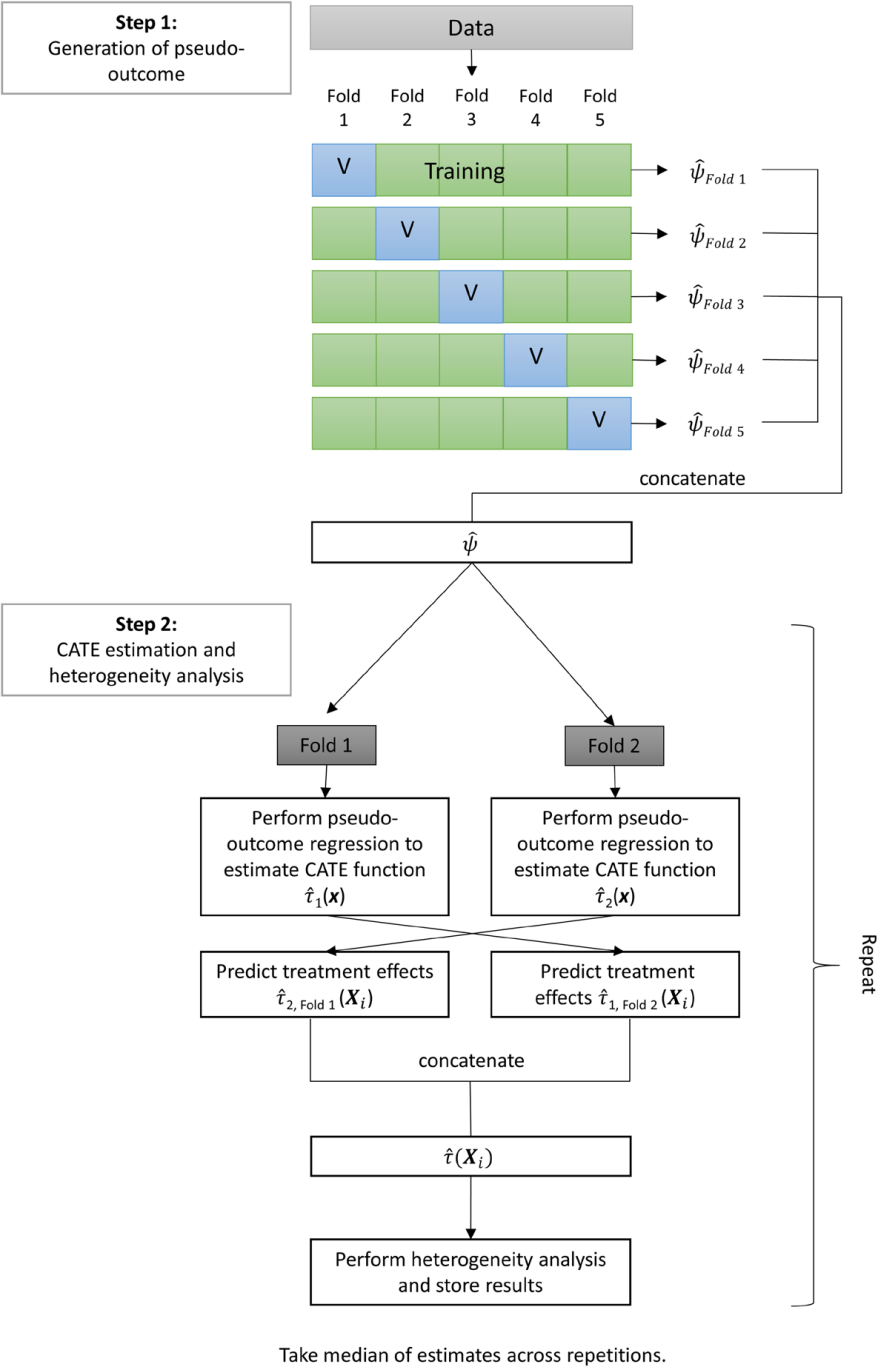
2-step cross-fitting procedure. *Note.* In this illustration, the first step uses 5-fold cross-fitting for generating the pseudo-outcomes and the second step uses (repeated) 2-fold cross-fitting for pseudo-outcome regression and heterogeneity analysis

**Obtaining valid inference** As stated above, it is important to use independent persons for fitting the CATE function and for performing inference on the estimated treatment effects in order to obtain valid results. When an independent test set is not available, one can use sample splitting. In case of the S-learner and the T-learner (see Figure 7 A), this means that in a first step, a (random) part of the sample is used to estimate the conditional mean function(s) as well the two nuisance functions that are needed for the global heterogeneity test and the GATES (i.e., ${\hat{\pi }}({\varvec{x}})$ and ${\hat{m}}({\varvec{x}})$). Then predictions are obtained for the other part of the sample and these are used for the heterogeneity analysis. To increase efficiency, one could use cross-fitting to obtain out-of-fold predictions for the whole sample (see Figure 7 B), such that all data is used in the heterogeneity analysis. Furthermore, because the results of the tests depend on the specific way the data was split, Chernozhukov et al. (2018b) suggested to repeat the sample splitting process multiple times (e.g., 100) and to aggregate the parameter estimates ($\beta _1$, $\beta _2$, $\gamma _k$, etc.), confidence intervals, and p-values by taking the medians across the repeated splits. This has the effect that the p-values account both for the estimation uncertainty and for the uncertainty induced by the sample splitting.

In case of the pseudo-outcome methods, one can include an additional split to prevent overfitting in the pseudo-outcome regression. To do so, one splits the sample into three folds, uses the first fold for estimating the nuisance functions, the second fold to estimate the CATE function ${\hat{\tau }}({\varvec{x}})$, and the third fold to perform the heterogeneity analysis on the predicted treatment effects. This sample splitting scheme is illustrated in Figure 7 C. However, with small sample sizes or when there are only few observations in one of the groups, as is the case in our illustrative example, splitting the data into three folds likely results in severe underfitting and loss in power. Therefore, following Jacob (2021) we used a two-step cross-fitting procedure (see Figure 8 in the appendix for a graphical illustration) that consisted of generating an out-of-fold pseudo-outcome for the full sample in a first step. In the second step, we used 10-fold cross-fitting for the estimation of the CATE function and the heterogeneity analysis, which was repeated 50 times. That is, in each of the 50 repetitions, we (i) obtained a CATE estimate ${\hat{\tau }}({\varvec{X}}_i)$ for each person *i*, whereby the function ${\hat{\tau }}({\varvec{x}})$ was estimated on a sub-sample that did not entail *i*, (ii) performed the analysis on these cross-fitted estimates, and (iii) stored the results. The final results were obtained by taking the median across the repetitions (see the supplementary material for the R code). We chose 50 repetitions based on the results of Jacob (2020) and 10 folds to have sufficient observations in the training folds to adequately learn the CATE function.^17^ However, we caution that this is a novel procedure and that simulation studies are required to show that it provides valid results and to compare it to alternative implementations of sample splitting and cross-fitting.

## Conclusion

Clinical psychologists are interested in finding the best possible treatment for patients. In this tutorial, we described different meta-learners that use off-the-shelf machine learning methods for estimating the CATE. Informally, a meta-learner specifies what to estimate in which order, but the researcher needs to decide upon the *how*, that is, which machine learning methods to use for estimation. While presenting descriptive statistics of the estimated CATE is informative in its own right, we also illustrated how the estimates can be used to further analyse treatment effect heterogeneity (i.e., to test whether there is significant heterogeneity, to test hypotheses regarding subgroup-specific CATEs, and to examine which covariates are associated with the underlying heterogeneity). We also pointed out how current popular practices in psychotherapy research fall under the meta-learner framework. Furthermore, we discussed the use of sample splitting and cross-fitting in order to prevent overfitting of the more complex meta-learners and to ensure valid results when making inference on heterogeneous treatment effects. As our descriptions have shown, meta-learners entail many researchers’ degrees of freedom, underlining the importance of transparency and the need for guidelines for best practices. However, despite these challenges, the high flexibility of meta-learners provides the tools for estimating the CATE with high accuracy and precision in a variety of data settings.

## Glossary

- **base-learner** refers to any machine learning method that is used within a meta-learner to solve a prediction task.
- **conditional independence assumption** is the assumption that conditional on the observed covariates, the potential outcomes of person *i* are independent from whether or not *i* receives treatment, that is, independent from how person *i* would respond to treatment. Formally, $A_i \perp \lbrace Y(0), Y(1) \rbrace \vert {\varvec{X}}_i$. In observational studies where persons self-select into treatment, this is a strong assumption since it rules out any unobserved confounding, and should be assessed carefully based on theoretical considerations and sensitivity analysis.
- **conditional mean method** refers to meta-learners that rely on estimating the conditional mean functions of the outcome only, i.e., that do not incorporate additional information such as the propensity score. Examples are the T-learner and the S-learner.
- **covariate imbalance** occurs when the treatment and control group differ in their covariate distributions. Propensity score methods aim to balance the distribution of covariates between the two groups in order to prevent that the treatment effect estimation is biased by group differences in the observed covariates. Strong covariate imbalance can result in (near) violations of the positivity assumption.
- **cross-fitting** is a sample splitting technique that separates the estimation of nuisance parameters from the estimation of the parameter of interest (e.g., the CATE).
- **cross-validation** is a sample splitting technique that uses separate subsamples for training the model and for evaluating the model’s performance. It is mainly used for hyperparameter tuning and for obtaining an realistic estimate of a model’s prediction error.
- **doubly-robustness** is a property of a causal estimator; an estimator is called doubly-robust when it remains consistent as long as either the propensity score or the conditional mean function(s) of the outcome are correctly specified.
- **hyperparameter** is a parameter whose value affects the training of the model. Thus, hyperparameters have to be specified a-priori, whereas the “normal” model parameters are learned during training. For example, in lasso regression the shrinkage parameter $\lambda$ is a hyperparameter: it affects how the model parameters (e.g., the coefficients $\beta$) are estimated (e.g., whether they are set to zero).
- **hyperparameter tuning** is the process of selecting a set of optimal hyperparameter values for a machine learning algorithm. Here, “optimal” refers to the predictive performance of the resulting model when used to predict the outcome for new observations (i.e., observations that are not used to train the model). The predictive performance is assessed via the loss of the algorithm. Hyperparameter tuning is often performed via cross-validation.
- **loss function** captures the deviation between a model’s predicted values and the true values. Machine learning algorithms build a predictive model by minimizing a given loss function, hence their predictive performance strongly depends upon the choice of loss function. For example, a common loss function for regression tasks is the mean squared error, MSE $= \frac{1}{n} \sum _{i=1}^{n} (Y_i - {\hat{Y}}_i)^2$, which measures the squared differences between the actual and the predicted values.
- **machine learning** is used synonymously to *supervised learning* in this tutorial. Supervised learning refers to any algorithm that uses data points with observed outcome values to build a predictive model, that is, to build a function that maps the observed outcome *Y* on the covariates ${\varvec{X}}$
- **meta-learner** is a meta-algorithm that breaks down the task of estimating the CATE into several prediction tasks, each of which can be solved using any machine learning method.
- **model stacking** refers to algorithms that combine several machine learning models into a new predictive model. The motivation is that different machine learning models have different strengths, and it is generally difficult to choose which one to use. Thus, model stacking aims to find combinations of (fitted) machine learning models that optimize the predictive performance. An example for a model stacking algorithm is the Super Learner.
- **model training** is synonymous to building a model; it is the process of applying a machine learning algorithm to training data, yielding a predictive model.
- **model tuning** *see* hyperparameter tuning.
- **nuisance parameter (nuisance function)** is any parameter (function) that is unspecified and has to be approximated in order to estimate or test hypotheses regarding the parameter of interest. In the case of meta-learners, the conditional mean functions or the propensity function are examples for nuisance functions: We are not interested in these functions themselves, but need to approximate them in order to estimate the CATE.
- **out-of-bag prediction** In a random forest, the out-of-bag prediction for a person *i* is the average prediction from the trees that do not contain *i* in their respective bootstrap sample.
- **overfitting** occurs when a model fits the training data too closely, and therefore does not generalize well to new data (i.e., fails to adequately predict the outcome for new observations that were not used for training the model).
- **positivity assumption** is the assumption that the propensity score is bounded away from 0 and 1, formally, $0< \pi ({\varvec{x}}) < 1$ for all possible covariate combinations ${\varvec{x}}$. This implies that for any possible combination of observed covariate values, there exist both treated and untreated persons. Also referred to as *sufficient common support* or *overlap* assumption.
- **propensity score** is the conditional probability of receiving treatment given the observed covariates. Formally, $\pi ({\varvec{x}}) = P(A_i=1 \vert {\varvec{X}}_i = {\varvec{x}}.$
- **pseudo-outcome** is an initial approximation of the CATE that is regressed onto the observed covariates in order to obtain a final CATE estimate.
- **pseudo-outcome method** refers to meta-learners that operate via a pseudo-outcome. Examples are the X-learner and the DR-learner.
- **R-loss** is a squared-error loss specifically designed to capture heterogeneous treatment effects while controlling for potential confounding. The R-loss is used by the R-learner as well as the causal forest.
- **regularization** refers to techniques that constrain a model’s complexity in order to avoid overfitting. This is achieved by including a penalty term in the loss function. For example, lasso regression minimizes the loss function
  $$\begin{aligned} L_{\text {lasso}} = \frac{1}{n} \sum _{i=1}^{n} (Y_i - {\hat{Y}}_i)^2 + \underbrace{\lambda \sum _{j=1}^{p} \vert \beta _j \vert }_{\text {penalty term}} \end{aligned}$$
  where ${\hat{Y}}_i = \beta _0 + \beta _1 X_{i1} + \ldots + \beta _p X_{ip}$ are the model’s predictive values and $\lambda \ge 0$. Adding the penalty term has the effect that large absolute coefficients can result in higher values of the loss function despite decreasing the errors $(Y_i - {\hat{Y}}_i)^2$, such that the algorithm seeks to find a good balance between the model’s complexity and predictive accuracy in the training data. $\lambda$ determines the degree of regularization, that is, how much the model’s coefficients are shrinked towards zero.
- **stable unit treatment value assumption (SUTVA)** assumes that for each person *i*, the observed outcome equals the potential outcome under the treatment level actually received, formally, $Y_i = Y_i(A_i)$. This entails that the treatment levels are well-defined and rules out any interference between persons.
- **Super Learner** is a variant of model stacking. Despite the similar name, it is *not* a meta-learner (but can be used as base-learner within meta-learners, for example).
- **supervised learning** *see* machine learning.
- **underfitting** occurs when a model fails to capture the underlying patterns in the data, such that it neither performs well on the training data nor generalizes to new data.

## Supporting information

Additional supporting information can be found online in the OSF project accompanying this article, see https://osf.io/t97xr/?view_only=9e047319bbc5431ea30f724fdeb60db3.

## Supplementary Information

Below is the link to the electronic supplementary material.

Supplementary file 1 (r 37 KB)

Supplementary file 2 (r 35 KB)

Supplementary file 3 (r 20 KB)

## Data Availability

The data that support the findings of this study are openly available from the Inter-university Consortium for Political and Social Research at https://www.icpsr.umich.edu/web/ICPSR/studies/21600?archive=ICPSR &q=21600#.

## Footnotes

1 For introductions to the causal tree, causal forest, causal BART, and causal boosting, we refer to Hu (2023), Jacob (2021), and Carnegie et al. (2019). Also, several other modified machine learning methods were proposed, including methods that rely on lasso regression (Qian & Murphy, 2011), support vector machines (Imai & Ratkovic, 2013), multivariate adaptive regression splines (Powers et al., 2018), neural networks (Johansson et al., 2016; Shalit et al., 2017; Schwab et al., 2018; Curth & van der Schaar, 2021), and deep kernel learning (Zhang et al., 2020).

2 This is called the stable unit treatment value assumption (SUTVA) in the causality literature and requires that the potential outcomes for a person *i* are not affected by whether other persons receive treatment or not (i.e., there are no spillover effects) and that there are no different versions of the treatment and control condition which lead to different potential outcomes (Imbens & Rubin, 2015).

3 These assumptions have many different names in the literature. Conditional independence is also called conditional exchangeability, conditional ignorability, no hidden bias, and selection on observables. The positivity assumption is also called overlap assumption or sufficient common support (Imbens, 2004; Hernan & Robins, 2020).

4 Strictly speaking, this is the definition of supervised learning, and we use the terms machine learning and supervised learning interchangeably in this article. Other forms of machine learning include semi-supervised, unsupervised, and reinforcement learning (see e.g., Burkov, 2020, for a definition of these terms).

5 The more hyperparameters are tuned, the slower the tuning process. However, not all hyperparameters are equally important, and often simulation studies found some hyperparameters to work well under their default values. Also, sometimes hyperparameters are interdependent such that it is possible to fix one hyperparameter to a specific value and only tune the other one given that value.

6 We tune the models following the recommendations of Boehmke and Greenwell (2019). When showing how the different meta-learners can be implemented in R below, we present simplified code which fits random forest models with default settings in order to ease readability. In the supplementary material, we provide the full code, including hyperparameter tuning. For pseudo-code representations of the meta-learners, see e.g., Okasa (2022).

7 More specifically, a pseudo-outcome is defined as an unbiased estimator of the CATE when computed with the *true* (rather than estimated) nuisance functions. That is, ${\mathbb {E}}[\psi _i \vert {\varvec{X}}_i = {\varvec{x}}] = \tau ({\varvec{x}})$, where $\psi _i$ denotes the pseudo-outcome when computed with the true nuisance functions. Hence, by regressing the estimated pseudo-outcome on the covariates ${\varvec{X}}_i$, one obtains an estimate of the CATE function, $\hat{{\mathbb {E}}}[\psi _i \vert {\varvec{X}}_i = {\varvec{x}}] = {\hat{\tau }}({\varvec{x}})$.

8 Typically, the estimated propensity score is used as a weighting function, but in principle, any weighting function that takes values in [0, 1] could be used for averaging the two estimates (Künzel et al., 2019).

9 Also, for minimizing the R-loss the R-Learner specifically requires machine learning methods that incorporate some form of regularization – that is, methods that penalize the complexity of the CATE by shrinking some parameters towards zero.

10 One possibility to enforce the coefficient of $A_i$ to remain in the model would be to use the general linear model as base-learner. However, as outlined in the introduction, this can result in bias when the functional form is misspecified and is not always feasible when the number of covariates is large.

11 To provide more intuition for the weighting, consider a data setting where there are many more persons in the control than in the treatment group, as in the illustrative example. Then ${\hat{\mu }}_1({\varvec{x}})$ is estimated with much greater uncertainty than ${\hat{\mu }}_0({\varvec{x}})$. Because ${\hat{\tau }}_0({\varvec{x}})$ relies on estimates from ${\hat{\mu }}_1({\varvec{x}})$ and ${\hat{\tau }}_1({\varvec{x}})$ on estimates from ${\hat{\mu }}_0({\varvec{x}})$, one can expect ${\hat{\tau }}_1({\varvec{x}})$ to be more accurate. By weighting ${\hat{\tau }}_1({\varvec{x}})$ with $1-{\hat{\pi }}({\varvec{x}})$ and ${\hat{\tau }}_0({\varvec{x}})$ with ${\hat{\pi }}({\varvec{x}})$, the X-learner gives more weight to the presumably more accurate ${\hat{\tau }}_1({\varvec{x}})$, since the propensity score ${\hat{\pi }}({\varvec{x}})$ is overall small when few persons were treated.

12 Setting extreme propensity scores to a less extreme value or discarding all persons with propensity scores outside of a certain range (see, e.g., Crump et al., 2008) are common strategies to deal with (near) violations of the positivity assumption. However, the choice of cutoff values is often arbitrary and such ad hoc modifications can change the meaning of the causal effect estimates (see, e.g., Li et al., 2019).

13 It would be interesting to directly compare the prediction performance of the different meta-learners (using an independent test set). However, this is difficult since the true CATE is unobservable. Nevertheless, Athey et al. (2020) proposed two measures for comparing the MSE of CATE estimators, which are based on a specific transformed outcome and on the R-loss.

14 Our definition of cross-fitting is also called ’combined approach’ in the literature (Jacob, 2020). However, there is also an ’averaging’ variant, which is defined as follows: After having used $S_1$ for nuisance function estimation and $S_2$ for pseudo-outcome regression, the roles are reversed and $S_2$ is used for nuisance function estimation and $S_1$ for pseudo-outcome regression. This results in two estimated CATE functions that each can be used to generate a prediction for the CATE of a person. These CATEs are then averaged to obtain the final CATE, ${\hat{\tau }}({\varvec{x}}) = \frac{1}{2} \left( {\hat{\tau }}_{S_1}({\varvec{x}}) + {\hat{\tau }}_{S_2}({\varvec{x}}) \right)$. Again, this 2-fold cross-fitting procedure can be extended to using *k* folds.

15 Currently, there is no standard approach to obtain a (valid) confidence interval for a single individual treatment effect. Künzel et al. (2019) evaluated several bootstrap procedures to obtain confidence intervals and found the different procedures to perform similar, but none provided the correct coverage. However, the authors investigated full-sample versions of the meta-learners, and estimating standard errors via bootstrapping might work better when using sample splitting within the meta-learners (Okasa, 2022; see also Jacob, 2021 for implementations of bootstrapping for meta-learners.). Also, in the special case where ordinary least squares regression (rather than a typical machine learning method) is used to obtain the CATE estimates in the last step of the pseudo-outcome methods, standard normality-based confidence intervals for the CATE estimates are valid. In the full code in the supplementary material, we show how this can be implemented in R.

16 In difference, ’regular’ group average treatment effects are average effects for groups that are defined by a (small) set of pre-chosen covariates (such as the CATE for old persons with a university degree).

17 As we have discussed above, the X-learner seems to work best in its full-sample version, which is why we did not use cross-fitting in the first step in our example (but we used the random forests’ OOB predictions). Also, we included the estimation of the propensity score $\pi ({\varvec{x}})$ and the conditional mean function $m({\varvec{x}})$ (which are needed for the computation of the CATE and/or the heterogeneity analysis) in the repeated 10-fold cross-fitting in the second step.
